# Waste Polymer and Lubricating Oil Used as Asphalt Rheological Modifiers

**DOI:** 10.3390/ma15113744

**Published:** 2022-05-24

**Authors:** Khalid Ahmed Owaid, Ammar Ahmed Hamdoon, Rand Raad Maty, Mohanad Yakdhan Saleh, M. A. Abdelzaher

**Affiliations:** 1 Department of Chemistry, College of Education for Pure Science, University of Mosul, Mosul 41002, Iraq; khalid.a.waid73@uomosul.edu.iq (K.A.O.); ammarhamdoon@uomosul.edu.iq (A.A.H.); mohanadalallaf@uomosul.edu.iq (M.Y.S.); 2 Department of Medical Laboratories Technologies, Al-Noor University College, Barttel 10008, Iraq; randd.raad@alnoor.edu.iq; 3 Environmental Science and Industrial Development Department, Faculty of Postgraduate Studies for Advanced Sciences, Beni-Suef University, Beni-Suef 62511, Egypt

**Keywords:** asphalt, rheological properties, polymers, ULO, microstructure

## Abstract

The hazards of plastic waste (PW) from polymers (e.g., polyethylene terephthalate (PET), high-density polyethylene (HDPE), low-density polyethylene (LDPE), polyvinyl chloride (PVC), nylon, polystyrene (PS), etc.), the mechanism of its spread in general, and its ubiquity in our daily lives as a continuously and/or frequently expelled product are a crisis of the twenty-first century, as reported by the United Nations in 2019, especially after the outbreak of the COVID-19 pandemic. This research included the process of modifying the rheological properties of asphalt to obtain asphalt suitable for use in a high-humidity atmosphere. The Iraqi climate is characterized by heat that reaches the point of harshness in the summer and coldness that falls below zero on some winter days. From this point of view, our recent study focuses mainly on making rheological and chemical modifications to asphalt using spent polymeric materials and used lubricating oils (ULO), thus achieving two important goals, namely obtaining asphalt with rheological properties resistant to the Iraqi atmosphere as well as eliminating both solid and liquid environmental pollutants. The microstructure and morphology of the designed patches were characterized using scanning electron microscopy (SEM) to indicate phase composition.

## 1. Introduction

Plastic poses a huge burden to the environment due to its recalcitrant nature, which makes it resistant to biodegradation, as plastic left in the same condition for a long period is a huge threat to the environment, plant life, and wildlife, as well as to people. Elastomers are a group of organic polymers comprising synthetic, semi-synthetic, or natural materials that can be folded and shaped into solid bodies [1,2]. Polymers are large-molecular-weight synthetic materials made of long chains consisting of carbon and other elements, such as hydrogen, chlorine, and nitrogen. Each unit in the chain is called a monomer, and it is a chemical substance that is produced from crude oil and gases. The demand for plastic has increased in the global market as an alternative to natural rubber, glass, wood, and other raw materials and metals, which may be attributed to the properties that plastics/polymers have, and as a result of these properties, these materials have many uses in many areas of our daily lives and as raw materials for different industries. The commercial and economic applications of plastics are a very unique “mixture” in terms of low cost, low toxicity, ease of processing, excellent thermal stability, and balance of physical properties [3,4,5,6,7]. In addition to that, with more cars, vans, and trucks on the road in Iraq, an increasing number of used lubricating oils (ULO) are generated. If not disposed of properly, used lubricating oils will threaten the environment and human health [8], especially waterways and soil, which might be polluted if ULOs are discharged. Meanwhile, with large-scale highway revamping and maintenance, there has been a higher demand for asphalt in road engineering. Because the fossil fuel reserve is decreasing around the world, alternative asphalt materials are badly needed in road engineering [9,10,11,12].

**Figure 1:**
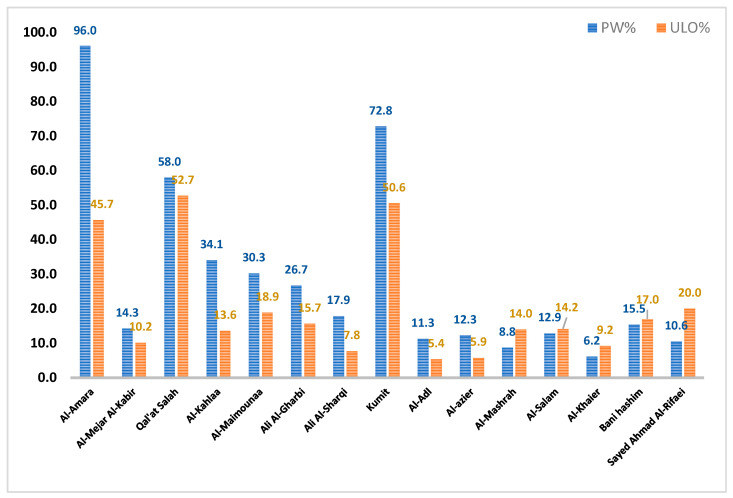
Accumulation of PW vs. ULO in tons throughout the Iraq’s governorates.

Asphalt is what remains after the vacuum distillation of crude oil, and has similar components to cured oil. Therefore, if oil could be used as a modifier for asphalt, its value would be much higher than just as a fuel [13]. According to the Iraqi Mobilization and Statistics Center, there are large amounts of polymer waste (PW) and used lubricant oil (ULO) that represent an environmental load, as they are unrecycled waste that can inflict negative drawbacks on the surrounding ecosystem. Figure 1 shows the accumulation of PW vs. ULO in tons. A mean PW of ~28.5 ton/year and a mean ULO of ~20.1 ton/year can be found throughout Iraq’s governorates [4,8].

Asphalt has certain properties of flexibility and fluidity and is an adhesive material, which qualifies it to be one of the most important materials included in the concrete mixtures used for paving streets [14,15,16]. To obtain good paving that is resistant to climatic conditions and heavy loads, thus increasing its operational life, the binder (asphalt) is modified by adding several materials to it to improve its properties [17]. Adding polymers to asphalt in certain proportions and in different ways contributes to improving its quality and increasing its hardness [16,18], thus reducing deformations, thatch, and creep that pavement is often exposed to. Recent studies deal with adding carbonation cake, which is waste from sugar factories, to the bitumen. After conducting tests of penetration, softening point, rotational viscosity, and dynamic shear on pure and modified asphalt, it was found that the carbonation cake had no positive or negative effect on rheological properties at the medium and high temperatures of the bitumen. From these experiments, promising results were obtained for the disposal of carbonation cake waste in different areas [19]. The modification of asphalt by adding reclaimed tire rubber under optimum conditions for the catalytic air blowing process resulted in a sample with excellent rheological properties [20]. In addition, modifying the characteristics of the asphalt using a mixture of used motor oils and polyethylene terephthalate shows that good results have been reached for the modified asphalt when compared to the non-modified asphalt [21]. Nano-silica was also added to the asphalt for certain proportions, and after conducting all required tests on both the original asphalt and the asphalt modified with nano silicate, it was found that the addition of nano silicate in the controlled mixture of asphalt improved its rheological properties well [22]. Another study focused on several points, namely, a review of studies related to the mechanical properties of asphalt mixtures containing bottom ash, as well as an indication of the effectiveness of this type of treatment of asphalt with bottom ash through testing. In addition, the results obtained in the evaluation of paving benefited from using the mechanistic–empirical pavement design guide (MEPDG) analysis. The results showed that asphalt mixtures that use bottom ash need a high percentage of asphalt due to the absorption factor and that the mixtures also had a lower mechanical coefficient compared to the control mixtures. The results of MEPDG indicated that these mixtures have a higher potential to corrode [23,24]. Matti, R et al. reported that they could modify the rheological properties of asphalt using microwave technology and by adding different polymers in certain proportions. Through the results of these tests that were carried out, good results were obtained, showing that the modified asphalt was better than the non-modified asphalt [25,26]. Furthermore, Han et al. studied the similarities and differences between the Marshall, Superpave, and GTM design methods by applying these methods to different asphalt mixtures, and the results showed that the GTM method leaves fewer voids and less bitumen and has higher heat and water stability compared to the other two methods. The resulting asphalt is characterized by a longer fatigue life compared to the other two methods and has a lower percentage of bitumen compared to the Marshall method. The volume parameters of this asphalt mixture can confirm specification very well, but the quality of the resulting asphalt is worse than the other two methods [27].

Moreover, Hamdoon et al. conducted a study that involved modifying the properties of asphalt by treating it with a mixture of polycarbonate and polymethyl methacrylate after cracking to obtain a low molecular weight, and the study included two paths, the first using a catalyst and the second using microwave rays with sulfur instead of the usual catalyst. The required tests were then conducted on the modified asphalt and showed good results for the modified asphalt, with the modified samples resisting climatic conditions [28]. The main objective of the present research is to evaluate the rheological characteristics of seven patches from PW and ULO on the modified bitumen’s binders.

## 2. Materials and Methods

### 2.1. Materials

The main raw precursors used in this laboratory investigation were asphalt type AH-70# (bitumen’s binders), a mixture of ethylene–vinyl acetate (copolymer as thermoplastic), used lubricating oils, and polyvinyl chloride. Ethylene–vinyl acetate (EVA) and polyvinyl chloride (PVC) were purchased from Sigma-Aldrich CO., LTD. (Baghdad, Iraq); the technical specifications are tabulated in Table 1. Asphalt type AH-70# was taken from a petroleum refinery (Northern Qayyarah oil field, North Mosul City, Iraq). Asphalt has a conventional composition, consisting of compounds of hydrogen and carbon with minor proportions of nitrogen, sulfur, and oxygen. ULO was sourced from a used vehicle’s lubricating oil. It is a synthetic material (fractions of petroleum oil distillates), a complex mixture of low and high (C_15_-C_50_)-molecular-weight aliphatic and aromatic hydrocarbons, lubrication additives, metals, and various organic and inorganic compounds, with viscosity at 1355 deg C, varying between 0.2 to 1.0 Pa·s, and a variable bulk density at 25 deg C. The sulfur used was obtained from the General Company for Sulfur Al-Mishraq, located south of the city of Mosul, Iraq. Its purity was close to 100%.

Five kilograms were collected from the asphalt then homogenized and dried at 105 °C for 24 h before usage. In addition, the physical properties of Iraqi binder bitumen vs. the standard testing measurements (JTG E20,2011) are detailed in Table 2 [29]. It is reported that the mean values for the bitumen binder’s softening point (%) are a high, as is the penetration (100 gm, 5 s, 25 °C). Properties of binder AS-70#, e.g., softening point, penetration, and ductility, are shown in Table 3.

### 2.2. Methods

**Figure 2:**
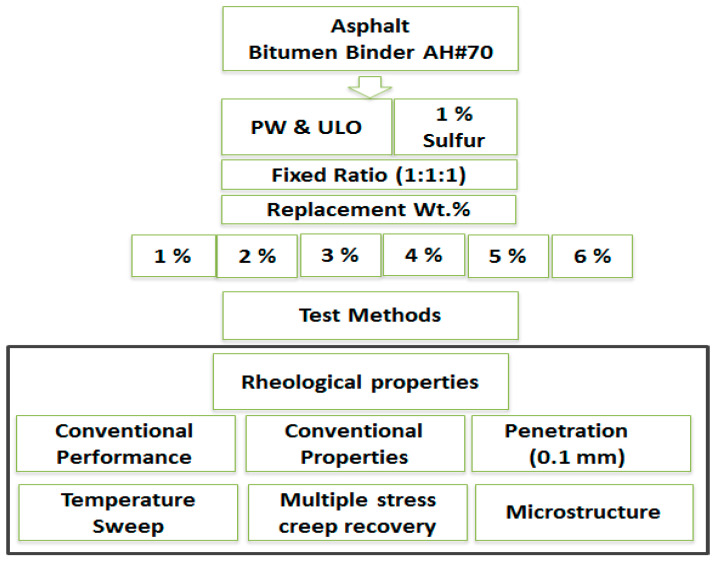
Scientific diagram of practical work.

Seven patches from PW and ULO-modified asphalt (bitumen’s binders) were prepared using an automatic mixer, as shown in Table 4. The AS matrix asphalt was kept at 180 °C, treated with a mixture of ethylene–vinyl acetate and spent car lubricating oils, as well as polyvinyl chloride where the PW and ULO were in a fixed ratio (1:1:1), with 1% sulfur by weight (as an addition), and replaced by 1, 2, 3, 4, 5, and 6% AH#70 asphalt with a 4000 rpm shear rate for 1 h. The temperature was kept at 180 °C for the duration of the experiment. Figure 2 shows the scientific diagram of the practical work. Microstructure analysis was performed using a field emission scanning electron microscopy (FE-SEM) model Hitachi S-4800, which is an advanced technology used to capture the microstructure image of materials. FE-SEM is typically performed in a high vacuum because gas molecules tend to disturb both the electron beam and the emitted secondary and backscattered electrons used for imaging.

## 3. Results and Discussion

### 3.1. The Conventional Performance

**Figure 3:**
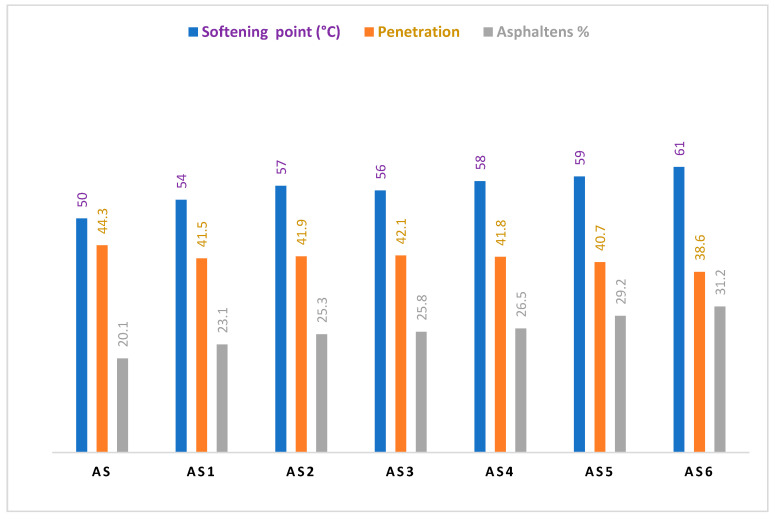
Conventional properties of PW and ULO-modified asphalt pastes.

**Figure 4:**
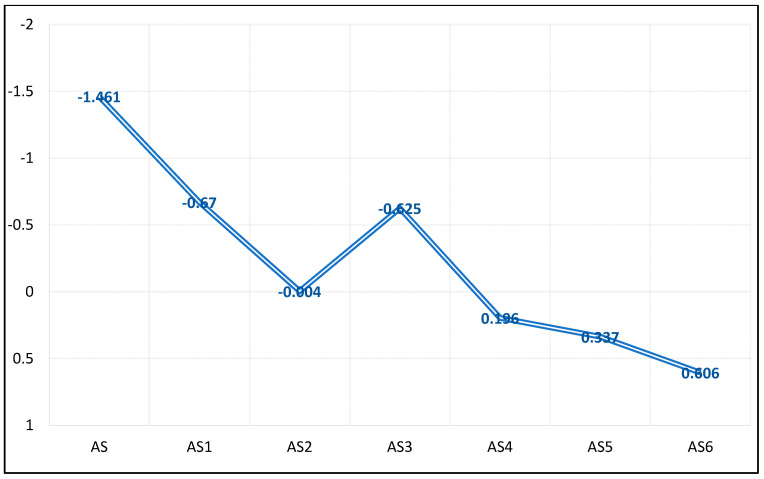
Penetration (0.1 mm) of PW and ULO-modified asphalt pastes.

It is evident from Table 5 and Figure 3, that the degree of ductility, penetration, softening point, and extension values for the PW and ULO-modified asphalt patches were within the limits of the standard specifications (JTGE20,2011) for asphalt paving shown in Table 1 and Table 2, up to a 5% Wt.% (AS-5) addition rate, accompanied by fulfilling values for both penetration and degree of ductility. Ductility values began to decrease at 5% by weight of the PW and ULO additive, but they were still within the acceptable values, as shown in Figure 3 [29,30,31]. While we note that the highest percentage, 6 Wt.% (AS-6), decreased the extension value significantly, and the values of ductility and extension were not within the standard specifications required for asphalt paving at this ratio, this might be attributed to the excess use of ULO for modification, as the grease may decrease the friction between the probe and asphalt, itself leading to a lesser extension effect for the PW and ULO additive, decreasing the density of the paste and increasing its porosity. As shown in Figure 4, this explains the penetration results of the modified asphalt patches in the following order: AS-2 > AS-3 < AS-4 had the lowest results, while AS-5 < AS-6 < AS-7 had the higher penetration results due to the extra addition of ULO, either from mobiles or biofuel, which indicated a negative performance when added to modified asphalt. AS#70 shows better penetration results than others.

### 3.2. Temperature Sweep

We note in Table 6 that the impact of the modified asphalt patches on the aging conditions of temperature sweep and oxidation is generally low. Aging conditions are attributed to the polymeric additive (PW), which improves the mechanical properties by increasing the strength and stress tolerance of the asphalt mix, reducing thermal cracking and increasing its resistance to the formation of grooves [32]. The rheological properties of modified asphalt patches with different additives are not affected by the conditions of aging, and their resistance to weather conditions is what qualifies them for use in the field of paving given the special nature of the weather conditions in Iraq, especially concerning the temperature differences in summer and winter [33,34,35]. AS-7 showed results outside of the standard, so it is excluded from the temp. sweep test. The dynamic viscosity at 135 deg C for asphalt was 0.2 to 1.4 Pa·s.

**Figure 5:**
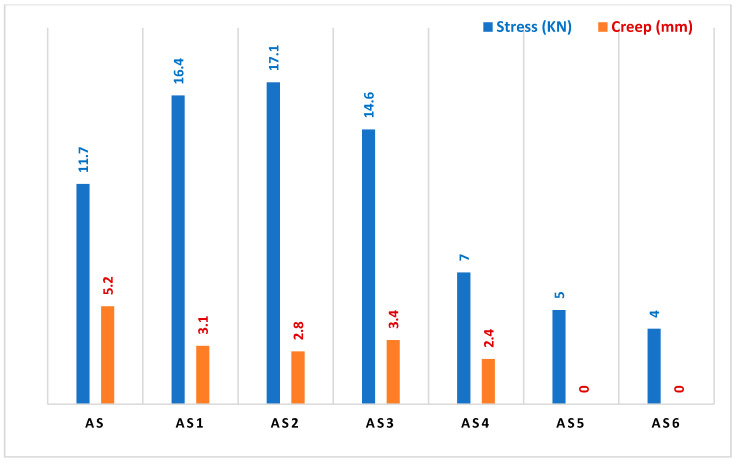
Multiple stress and creep recovery of PW and ULO-modified asphalt samples.

Moreover, as clearly seen from Table 7 (the multiple stress creep recovery of PW and ULO-modified asphalt pastes), all of the modified asphalt patches possessed better stress properties than the original asphalt if they were used as asphalt paving. We note from these results that the stability values of the modified asphalt patches were much better than the stability value of the original asphalt, even though the multiple stress creep recovery value of the original asphalt was within the standard specifications of asphalt paving [29]; however, we also note the high stress values of the modified models, which is a good indication of the ability of the paving to resist deformation resulting from a road being exposed to repeated transportation loads. Figure 5 proves that when stress increased, creep decreased. The stress value was lower than in the original asphalt, which makes it more resistant and stable with regard to creep when the road is exposed to repeated transportation loads; this indicates that the mixture has a small void ratio (MQ), which explains the exposure of many of the roads paved with this type of asphalt to creep and stress.

### 3.3. Morphology and Microstructure

**Figure 6:**
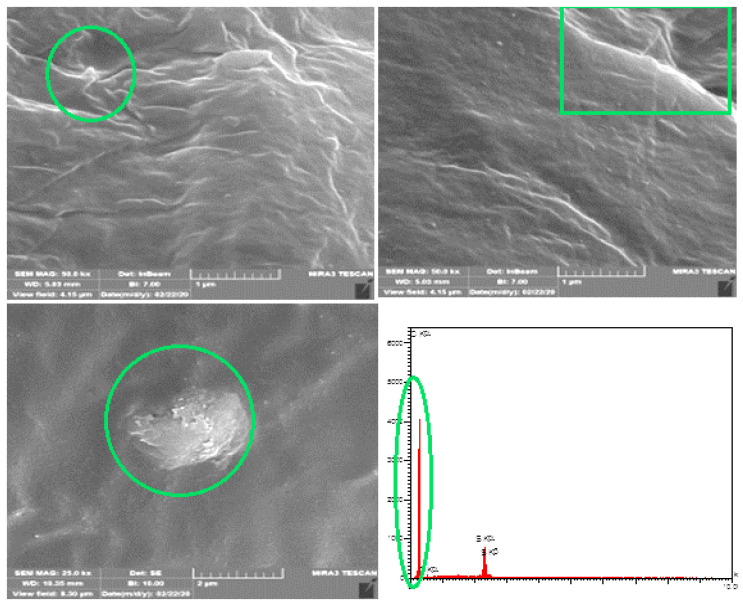
FE-SEM and EDX for asphalt (as received).

Figure 6 reports the results of the field emission scanning electron microscopy (FE-SEM) for AS#70 (asphalt sample as received). The differences in quality of asphalts depend on their crude oil sources because different crude oils have different chemical compositions, especially with regard to carbon (C) and hydrogen (H) content. AS#0 is a durable asphalt, as it possesses the physical properties necessary to produce the desired initial product performance properties and is resistant to changes in its physical properties during long-term, in-service environmental aging. AS#70 durability is determined by the physical properties of the asphalt, which are determined directly by chemical composition, as indicated by the green circle representing hydrocarbons, provided by EDX analysis [36,37].

**Figure 7:**
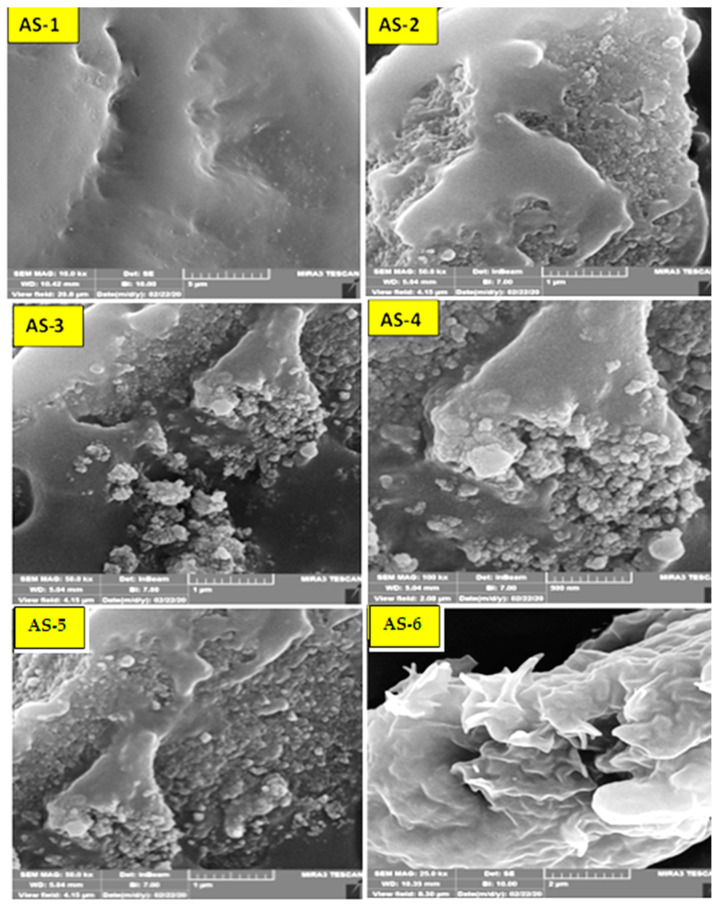
FE-SEM for PW and ULO-modified asphalt samples.

**Figure 8:**
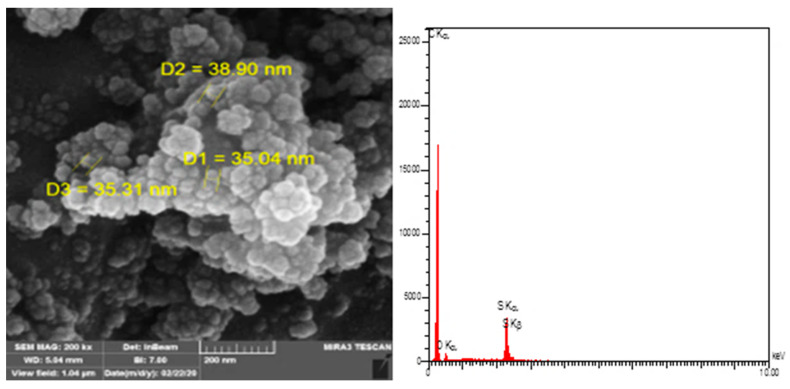
FE-SEM and EDX for AS-3 patch.

Figure 7 reports the results of the field emission scanning electron microscopy (FE-SEM) for PW and ULO-modified asphalt samples patches (AS-2, AS-3, AS-4, AS-5, AS-6, and AS-7), obtained by modifying asphalt with the polymeric mixture. The addition of polymer to the asphalt patches increased the complexity of the internal microstructure of the hub asphalt in all pastes and its effect on the rheological and morphological properties of the asphalt. This is because the shape and rheological properties of the hub asphalt were affected by the mutual effects of the polymer and asphalt. We can see in these images that the role of the added polymer is clear in changing the microstructure of the asphalt, as is its effect on the rheological and morphological properties of the polymer-centered asphalt. Its contents are carbon, sulfur, and oxygen [38,39,40]. The AS-3 patch sample shows that it had better physical properties than both the other patches and the native sample, and this may be attributed to the high surface area of PW powder, ranging from 35–38 nm. AS-3 has a 2.00 wt.% from PW and ULO replacement by a fixed ratio (1:1:1), which represents the optimal replacement ratio of waste to asphalt, as shown in Figure 8.

## 4. Conclusions

Material sustainability and solid-waste recycling are the main objectives of our research work. PW and ULO were used to investigate each binder’s rheological properties, establishing their chemical, microstructural, and mechanical relationship in order to detect basic regeneration mechanisms. We concluded that polymer waste and ULO might be used to improve the characteristics of high temperature stability and creep resistance in asphalt materials (AS#70). Seven patches of binder modifier were tested using different ratios. The local application of waste polymer can enhance road and highway construction engineering, promoting environmental protection and a sustainable development vision for road engineering in Iraq. The chemical compositional changes of the binders were consistent with rheological and microscopic analysis; in addition, the application of PW and ULO led to an increase in viscosity and created visible fibrous microstructures. We recommend that the AS-3 patch should have a 2.00 wt.% from PW and ULO replacement by a fixed ratio (1:1:1), which represents the optimal replacement ratio dosage of waste to binder. In addition, this proves the hypothesis that microstructure is intimately associated with binder mix composition and rheological properties, and that it is not just an unrelated, superficial phenomenon. Additional research is needed in order to reach sustainability in raw materials, as asphalt is a highly consumable material.

## Figures and Tables

**Table 1:** Technical specification of ethylene–vinyl acetate and polyvinyl chloride (as received).

Specification	Ethylene–Vinyl Acetate (C_6_H_10_O_2_)	Polyvinyl Chloride (CH_2_CHCl)_n_
Molecular weight	86.09	233.00
Bulk density	0.93 ± 0.02	0.50 ± 0.02
Specific gravity	2.0	1.4
Degree of polymerization	75–95%.	1000 ± 50
Melting point °C	88	-
K-Value	-	66

**Table 2:** Rheological properties of Iraqi paving asphalt vs. the standard testing measurements (JTG E20,2011).

Rheological Properties	Iraqi Paving			The Stander Testing Measurements (JTG E20,2011) [29]		
	Minimum	Maximum	Mean	Minimum	Maximum	Mean
Softening point (%)	54	60	60	54	65	60
Penetration (100 gm, 5 s, 25 °C)	40	50	43	20	40	30
Degree of Ductility (cm 25 °C)	10	-	11	15	-	15

**Table 3:** Properties of bitumen binder AH#70.

Rheological Properties	Minimu	Maximum	Mean
Softening point (°C)	57	66	60
Penetration (100 gm 5 s 25 °C)	18	40	28
Degree of Ductility (cm, 25 °C)	10	-	11

**Table 4:** Mix composition of PW and ULO-modified asphalt patches.

Mix Title	Mix Composition			
	AH#70 Asphalt by Weight (%)	PW and ULO Replacement by a Fixed Ratio (1:1:1)	Sulfur Addition by Fixed Weight (%)	Temp.
AS	100.0	0.0	1.0	180 °C
AS-1	99.0	1.0		
AS-2	98.0	2.0		
AS-3	97.0	3.0		
AS-4	96.0	4.0		
AS-5	95.0	5.0		
AS-6	94.0	6.0		

**Table 5:** Conventional properties of PW and ULO-modified asphalt pastes.

Mix Title	Conventional Properties				
	Ductility (cm)	Softening Point	Penetration	Penetration Index	Asphaltens %
AS	>150	50	44.3	−1.461	20.1
AS-1	>150	54	41.5	−0.670	23.1
AS-2	>150	57	41.9	−0.004	25.3
AS-3	>150	56	42.1	−0.625	25.8
AS-4	>150	58	41.8	+0.196	26.5
AS-5	133	59	40.7	+0.337	29.2
AS-6	67	65	37.3	+1.032	31.2

**Table 6:** Temperature sweep of PW and ULO-modified asphalt pastes.

Mix Title	Aging of Temp. Sweep	Rheological Properties				
		Ductility (cm)	Softening Point	Penetration	Penetration Index	Weight Loss
AS	Before	>150	50	44.3	−1.461	-
	After	>150	54	42.4	−0.618	0.062
AS-1	Before	>150	57	41.9	−0.004	-
	After	>150	58	41.4	+0.174	0.041
AS-2	Before	>150	57	42.1	−0.625	-
	After	>150	58	41.9	−0.004	0.039
AS-3	Before	>150	56	42.1	−0.625	-
	After	>150	58	41.6	+0.185	0.037
AS-4	Before	>150	55	40.8	−0.615	-
	After	>150	58	40.3	+0.144	0.031
AS-6	Before	>150	52	39.7	−0.612	-
	After	>150	57	40.1	+0.135	0.030

**Table 7:** Multiple stress and creep recovery of PW and ULO-modified asphalt pastes.

Mix Title	Multiple Stress Creep Recovery Test			
	Asphalt	Stability (KN)	Crawling (mm)	MQ
AS	4.5	11.7	5.20	2.25
AS-1		16.4	3.10	5.29
AS-2		11.7	2.80	6.10
AS-3		14.6	3.40	4.29
AS-4		7.0	2.4	2.91
AS-5		5.0	0.0	0.0
AS-6		4.0	0.0	0.0
AS *		7.0 (minimum)	2.40	3.50

* As: Sample Mean.

## References

[1] Chen X., Yan N. (2020). A brief overview of renewable plastics. Mater. Today Sustain..

[2] Chawla K., Singh R., Singh J. (2020). Segregation and Recycling of Plastic Solid Waste: A Review. Advances in Materials Science and Engineering.

[3] Ojogbo E., Ogunsona E.O., Mekonnen T.H. (2020). Chemical and physical modifications of starch for renewable polymeric materials. Mater. Today Sustain..

[4] Saleh H., Al-Kahlidi M.M.A., Abulridha H.A., Banoon S.R., Abdelzaher M.A. (2021). Current Situation and Future Prospects for Plastic Waste in Maysan Governorate: Effects and Treatment During the COVID-19 Pandemic. Egypt. J. Chem..

[5] Rahimi A., García J.M. (2017). Chemical recycling of waste plastics for new materials production. Nat. Rev. Chem..

[6] Kumar S., Panda A.K., Singh R.K. (2011). A review on tertiary recycling of high-density polyethylene to fuel. Resour. Conserv. Recycl..

[7] Sharuddin S.D.A., Abnisa F., Daud W.M.A., Aroua M.K. (2016). A review on pyrolysis of plastic wastes. Energy Convers. Manag..

[8] Abbas R., Shehata N., Mohamed E.A., Salah H., Abdelzaher M. (2021). Environmental safe disposal of cement kiln dust for the production of geopolymers. Egypt. J. Chem..

[9] Singh P., Tophel A., Swamy A.K. (2017). Properties of asphalt binder and asphalt concrete containing waste polyethylene. Pet. Sci. Technol..

[10] Singh P., Swamy A.K. (2019). Effect of aging level on viscoelastic properties of asphalt binder containing waste polyethylene. Int. J. Sustain. Eng..

[11] Tantawy M.A., El-Roudi A.M., Abdalla E.M., Abdelzaher M.A. (2013). Fire resistance of sewage sludge ash blended cement pastes. J. Eng..

[12] Cooper S.B., Mohammad L.N., Elseifi M.A. (2017). Laboratory performance of asphalt mixtures containing recycled asphalt shingles and re-refined engine oil bottoms. J. Mater. Civ. Eng..

[13] Rabeea M.A., Zaidan T.A., Ayfan A.H., Younis A.A. (2020). High porosity activated carbon synthesis using asphaltene particles. Carbon Lett..

[14] Klimisch H.J., Andreae M., Tillman U. (2003). Robust Summary of Information.

[15] Bulatović V.O., Rek V., Marković K.J. (2012). Polymer modified bitumen. Mater. Res. Innov..

[16] Shbeeb M.T. (2007). The use of polyethylene in hot asphalt mixtures. Am. J. Appl. Sci..

[17] Robinson H. (2005). Polymers in Asphalt.

[18] Elkhouly H.I., Abdelzaher M.A., El-Kattan I.M. (2021). Experimental and Modeling Investigation of Physicomechanical Properties and Firing Resistivity of Cement Pastes Incorporation of Micro-Date Seed Waste. Iran. J. Sci. Technol. Trans. Civ. Eng..

[19] Hussein A.A., Hamdoon A.A. (2021). The Use of a Mixture (Spent Lubricating Oils: Rubber) and Catalytic Air Blowing Process in the Rheological Modification of Asphalt. Adv. Mech..

[20] Al-Azzawi A., Hamdoon A. (2020). Mechanical performance study of asphalt by waste plastics and waste engine oil. AIP Conference Proceedings.

[21] Yao H., You Z., Li L., Lee C.H., Wingard D., Yap Y.K., Shi X., Goh S.W. (2013). Rheological Properties and Chemical Bonding of Asphalt Modified with Nanosilica. J. Mater. Civ. Eng..

[22] Goh S.W., You Z. (2008). A preliminary study of the mechanical properties of asphalt mixture containing bottom ash. Can. J. Civ. Eng..

[23] Abdelzaher M.A. (2021). Experiential investigation on the effect of heavy fuel oil substitution by high sulfur petcoke on the physico-mechanical features and microstructure of white cement composites. Eng. Res. Express.

[24] Matti R.R., Owaid K.A. (2020). Rheological modifications of the asphalt-polymer system using microwave technology. J. Educ. Sci..

[25] El-Kattan I.M., Abdelzaher M.A., Farghali A.A. (2020). Positive impact of ultra fine-ceramic waste on the physico-mechanical features and microstructure of white cement pastes composites. J. Mater. Res. Technol..

[26] Han D., Wei L., Zhang J. (2016). Experimental study on performance of asphalt mixture designed by different method. Procedia Eng..

[27] Shirini B., Imaninasab R. (2016). Performance evaluation of rubberized and SBS modified porous asphalt mixtures. Constr. Build. Mater..

[28] (2011). Standard Test Methods of Asphalt and Asphalt Mixtures for Highway Engineering.

[29] (2019). Standard Method of Test for Multiple Stress Creep Recovery (MSCR) Test of Asphalt Binder Using a Dynamic Shear Rheometer (DSR).

[30] Wróbel M., Woszuk A., Ratajczak M., Franus W. (2021). Properties of reclaimed asphalt pavement mixture with organic rejuvenator. Constr. Build. Mater..

[31] McDaniel R.S., Anderson R.M. (2001). Recommended Use of Reclaimed Asphalt Pavement in the Superpave Mix Design Method: Technician’s Manual (No. Project D9-12 FY’97).

[32] West R., Rodezno C., Julian G., Prowell D. (2014). Engineering Properties and Field Performance of Warm Mix Asphalt Technologies.

[33] Hicks R.G., Tighe S., Cheng D. (2012). Rubber Modified Asphalt Technical Manual. Proceedings.

[34] Iqbal M., Hussain A., Khattak A., Ahmad K. (2020). Improving the Aging Resistance of Asphalt by Addition of Polyethylene and Sulphur. Civ. Eng. J..

[35] Petersen J.C. (2000). Chemical composition of asphalt as related to asphalt durability. Developments in Petroleum Science.

[36] Balboul B.A., Abdelzaher M., Hamouda A.S., Zaki A.H. (2019). Nano titania combined with micro silica reinforced limestone cement: Physico-mechanical investigation. Egypt. J. Chem..

[37] Abdelzaher M.A., Hamouda A.S., Ismail I.M., El-Sheikh M.A. (2018). Nano titania reinforced limestone cement: Physico-mechanical investgation. Key Engineering Materials.

[38] Shawkey M.A., Abdelzaher M.A., Mahmoud H.M., Rashad M.M. (2021). Monitoring of acoustic emission behaviour during early-age cement paste hydration. Proceedings of the IOP Conference Series: Materials Science and Engineering.

[39] Abdelzaher M.A., Shehata N. (2022). Hydration and synergistic features of nanosilica-blended high alkaline white cement pastes composites. Appl. Nanosci..

[40] Kunanusont N., Sangpetngam B., Somwangthanaroj A. (2021). Asphalt Incorporation with Ethylene Vinyl Acetate (EVA) Copolymer and Natural Rubber (NR) Thermoplastic Vulcanizates (TPVs): Effects of TPV Gel Content on Physical and Rheological Properties. Polymers.

